# Supplementary material for: A critical role for HNF4α in polymicrobial sepsis-associated metabolic reprogramming and death
Source: EMBO Mol Med. 2024 Sep 11;16(10):13. doi: 10.1038/s44321-024-00130-1 (PMC11473810; doi:10.1038/s44321-024-00130-1)
Supplement: Supplementary file 1 — Appendix [file 44321_2024_130_MOESM1_ESM.pdf]

Table of contents

Appendix Figures

Figure S1: Many undifferential HNF4α ChIP-Seq & ATAC-Seq regions function in hepatic metabolism. p2

Figure S2: Increased hepatic steatosis and ALT in Hnf4a$^{Liver-i-KO}$ mice. p3

Figure S3: HNF4α agonist NCT improves hepatic steatosis, plasma ALT levels and hepatic acute phase response in sepsis. p4-5

Appendix Tables

Table S1: Pathway analysis HNF4α ChIP-Seq 8h after CLP. p6

Table S2: Pathway analysis HNF4α ChIP-Seq 24h after CLP. p7

Table S3: Pathway analysis ATAC-Seq 8h after CLP. p8

Table S4: Pathway analysis ATAC-Seq 24h after CLP. p9

Table S5: Pathway analysis IL6 Hnf4a$^{Liver-i-KO}$ RNA-Seq. p10

Table S6: List of primers used for qPCR analysis p11

Table S7: List of primers used for ChIP-qPCR analysis p11

A.

HOMER analysis: unchanged HNF4α binding sites in sham and CLP

Rank	Motif	Name	P-value
1	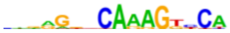	HNF4a	10^{-1470}
2	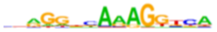	PPARa	10^{-708}
3	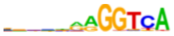	COUP-TFII	10^{-524}
4	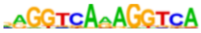	TR4	10^{-492}
5	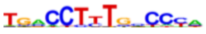	PPARE	10^{-467}
6	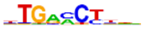	RARa	10^{-464}
7	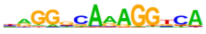	RXR	10^{-422}
8	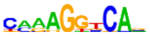	Erra	10^{-417}
9	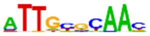	CEBP	10^{-392}
10	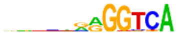	EAR2	10^{-365}

B.

Metascape pathway analysis: genes nearby unchanged HNF4α binding sites in sham and CLP with unchanged expression 8h after CLP

Description	P-value
TCA cycle and respiratory electron transport	$10^{-16.50}$
Protein catabolism	$10^{-14.88}$
Monocarboxylic acid metabolism	$10^{-13.17}$
Stress response	$10^{-12.71}$
Amide metabolism	$10^{-11.29}$
Carbon metabolism	$10^{-11.28}$
Sulfur compound metabolism	$10^{-10.45}$
Lipid metabolism	$10^{-9.80}$
Antigen processing: ubiquitination & proteasome degradation	$10^{-9.58}$
Golgi vesicle transport	$10^{-9.26}$

C.

HOMER analysis: open chromatin regions unchanged in sham and CLP

Rank	Motif	Name	P-value
1	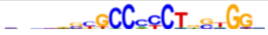	BORIS	10^{-26}
2	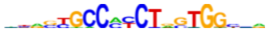	CTCF	10^{-22}
3	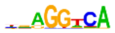	THRb	10^{-18}
4	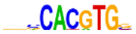	c-Myc	10^{-15}
5	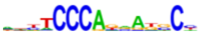	ZNF143|STAF	10^{-15}
6	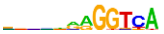	COUP-TFII	10^{-14}
7	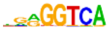	COUP-TFII	10^{-13}
8	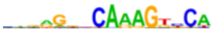	HNF4a	10^{-12}
9	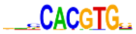	n-Myc	10^{-12}
10	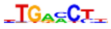	RARa	10^{-12}

D.

Metascape pathway analysis: genes within unchanged open chromatin regions in sham and CLP and unchanged expression 8h after CLP

Description	P-value
TCA cycle	$10^{-8.62}$
Carbohydrate derivative biosynthesis	$10^{-7.76}$
TCA cycle and respiratory electron transport	$10^{-7.37}$
Glycosyl compound metabolism	$10^{-7.33}$
Golgi vesicle transport	$10^{-7.30}$
RNA metabolism	$10^{-7.06}$
Intracellular protein transport	10^{-7}
Lipid metabolism	10^{-7}
Membrane localization	$10^{-6.69}$
Vesicle-mediated transport	$10^{-6.63}$

Appendix Figure S1. Many undifferential HNF4α ChIP-Seq & ATAC-Seq regions function in hepatic metabolism.

(A-B) HNF4α ChIP-Seq in liver, 8h after sham or CLP. n=4/group (biological replicates). **(A)** HOMER transcription factor motif enrichment 200 bp centered on the center of all undifferential peaks (Padj > 0.05). **(B)** Metascape pathway analysis of unchanged (Padj > 0.05 and -0.5 < LFC < 0.5) genes 8h after CLP associated with undifferential (Padj > 0.05) HNF4α binding sites. **(C-D)** ATAC-Seq in liver, 8h after sham or CLP. n=4/group (biological replicates). **(C)** HOMER transcription factor motif enrichment 200 bp centered on the center of all undifferential peaks (Padj > 0.05). **(D)** Metascape pathway analysis of unchanged (Padj > 0.05 and -0.5 < LFC < 0.5) genes 8h afer CLP associated with undifferential (Padj > 0.05) open chromatin sites.

P-values derived from Fisher's exact test (Hypergeometric test). For differential analysis, P-values were calculated from DESeq2 (Wald test).

A.

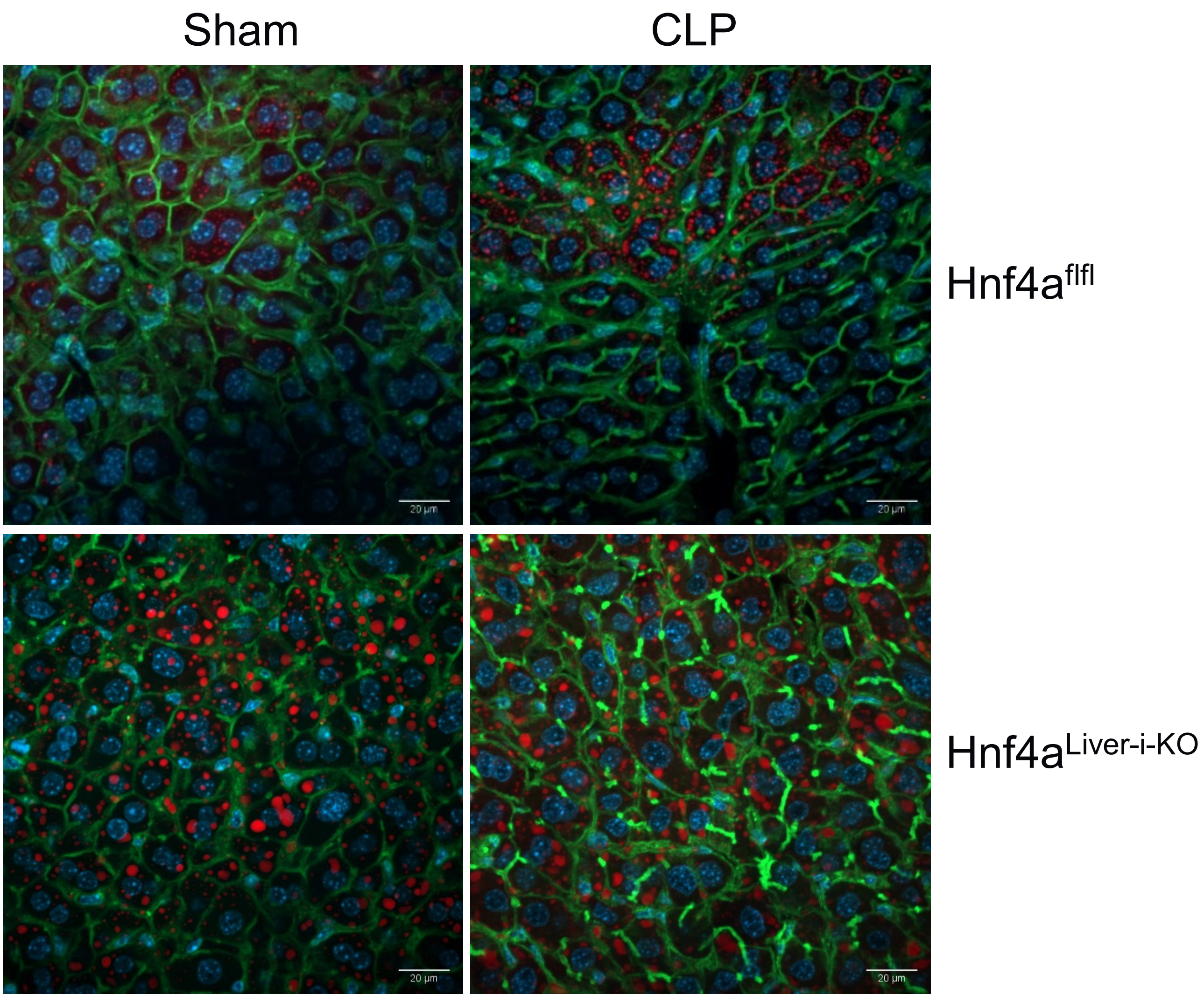

B.

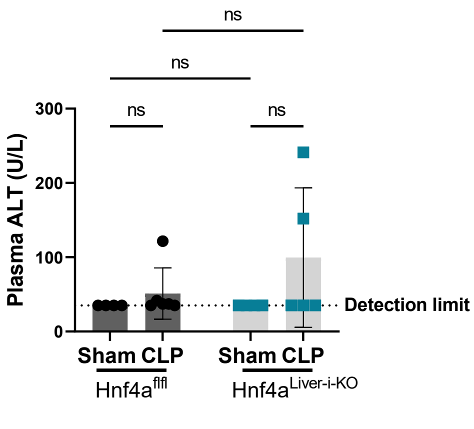

Appendix Figure S2. Increased hepatic steatosis and ALT in Hnf4a$^{Liver\text{-}i\text{-}KO}$ mice.

Hnf4a$^{Liver\text{-}i\text{-}KO}$ and Hnf4a^{flfl} mice were *i.p.* injected with tamoxifen on five consecutive days. Three days later, sham or CLP was performed, and liver and blood were isolated 8h and 24h later. **(A)** Immunofluorescent images of liver 8h after CLP stained with Actin-stain (green), DAPI (blue) and LipidTox (red), with 20x magnification. Z-stacks were generated in about 10 regions. White scale bar = 20 µm. n=31 (13 female, 18 male). **(B)** Plasma alanine aminotransferase (ALT) levels 24h after CLP. n=17-19 (7 female, 10-12 male).

Bars: mean±SD. Each dot represents a single biological replicate. P-values were analysed with two-way ANOVA. ns: nonsignificant.

A.

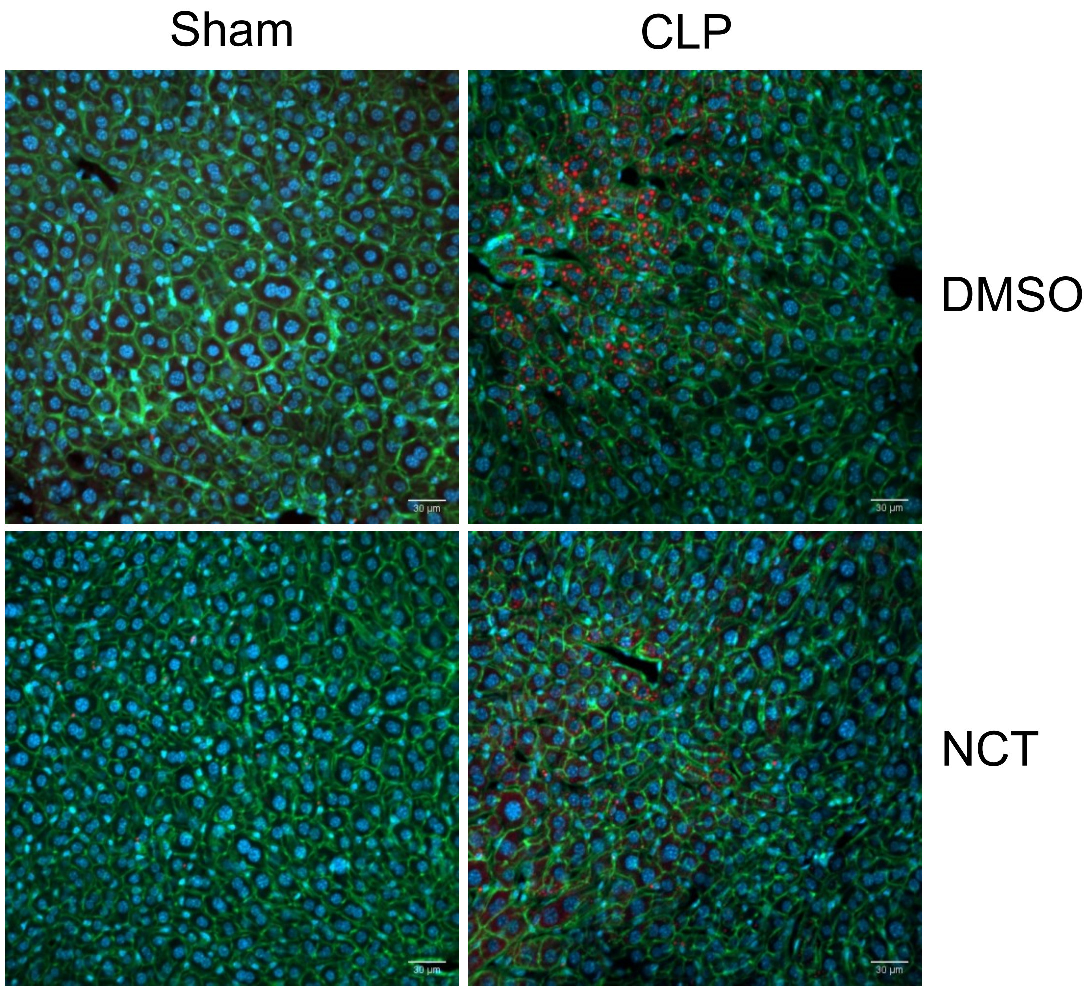
Sham
CLP
DMSO
NCT
30 µm

B.

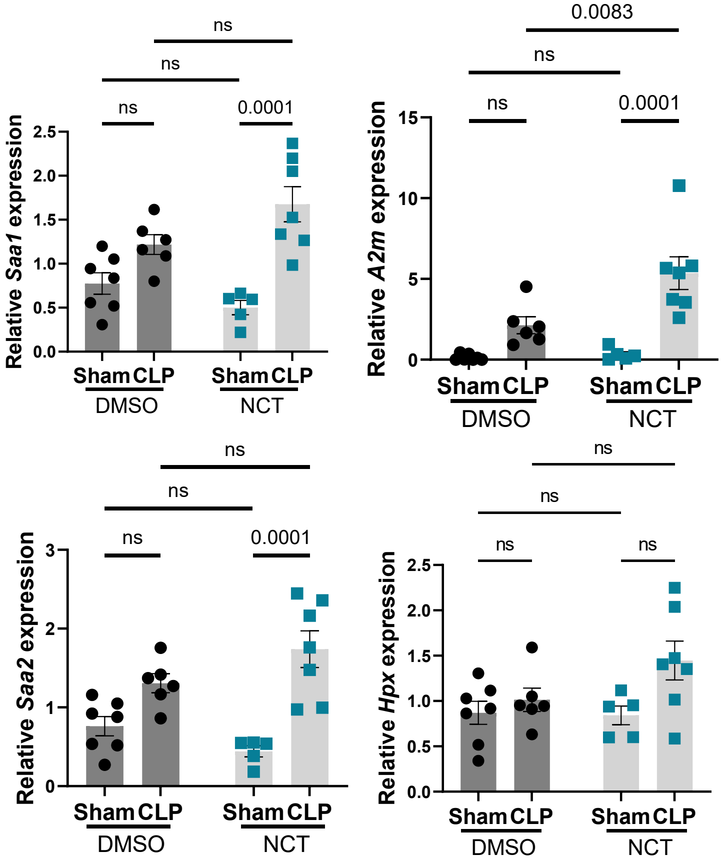
Relative Saa1 expression
ns
ns
ns
0.0001
Sham CLP
DMSO
Sham CLP
NCT
Relative A2m expression
0.0083
ns
ns
0.0001
Relative Saa2 expression
Relative Hpx expression

C.

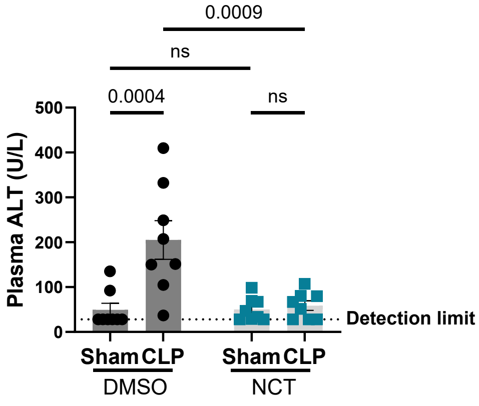
Plasma ALT (U/L)
0.0009
ns
0.0004
ns
Detection limit
Sham CLP
DMSO
Sham CLP
NCT

Appendix Figure S3. HNF4α agonist NCT improves hepatic steatosis, plasma ALT levels and hepatic acute phase response in sepsis.

Mice were *i.p.* injected with NCT (200 mg/kg) or DMSO twice daily for 6 days, subjected to sham or CLP on day 7, and liver and blood were isolated 24h later. n=3-8/group. **(A)** Immunofluorescent images of liver stained with Actin-stain (green), DAPI (blue) and LipidTox (red), with 20x magnification. Z-stacks were generated in about 10 regions. White scale bar = 30 µm. **(B)** RT-qPCR liver mRNA expression of *Saa1*, *Saa2*, *A2m* and *Hpx* relative to *Hprt* and *Rpl*. **(C)** Plasma alanine aminotransferase (ALT) levels 24h after CLP.

Bars: mean±SEM. Each dot represents a single biological replicate. P-values were analysed with two-way ANOVA. ns: nonsignificant.

Appendix Table S1. Pathway analysis HNF4α ChIP-Seq 8h after CLP.

Metascape pathway analysis: genes nearby sham-specific HNF4α binding sites with decreased expression 8h after CLP	
**Description**	**P-value**
**Lipid metabolism**	$10^{-4.66}$
ER signaling	$10^{-4.31}$
Regulation of intracellular steroid hormone receptor signaling	$10^{-4.21}$
**Regulation lipid metabolism**	$10^{-4.15}$
Epithelial cell differentiation	$10^{-4.10}$
Regulation vitamin D receptor signaling	$10^{-3.90}$
**Bile duct development**	$10^{-3.90}$
Rho GTPase signaling	$10^{-3.85}$
Negative regulation cell proliferation	$10^{-3.74}$
Aspartate & asparagine metabolism	$10^{-3.62}$

Metascape pathway analysis: genes nearby CLP-specific HNF4α binding sites with increased expression 8h after CLP	
**Description**	**P-value**
Response to peptide	$10^{-4.74}$
**Response to growth factor**	$10^{-4.65}$
Leukocyte homeostasis	$10^{-4.48}$
Regulation cellular catabolic process	$10^{-4.39}$
**Adipogenesis**	$10^{-4.31}$
Positive regulation apoptosis	$10^{-4.25}$
Regulation signaling receptor activity	$10^{-4.05}$
Regulation fibroblast migration	$10^{-3.96}$
Bacterial response	$10^{-3.82}$
Regulation cytokine production	$10^{-3.62}$

Table S1 displays Metascape pathway analysis of downregulated (Padj < 0.05 and LFC < 0) or upregulated (Padj < 0.05 and LFC > 0) genes 8h after CLP associated with sham-specific (Padj < 0.05 and LFC < 0) or CLP-specific (Padj < 0.05 and LFC > 0) HNF4α binding sites, respectively, in liver 8h after sham or CLP. P-values derived from Fisher's exact test (Hypergeometric test). For differential analysis, P-values were calculated from DESeq2 (Wald test).

Related to figure 2.

Appendix Table S2. Pathway analysis HNF4α ChIP-Seq 24h after CLP.

Metascape pathway analysis: genes nearby sham-specific HNF4α binding sites with decreased expression 24h after CLP	
**Description**	**P-value**
Lipid metabolism	$10^{-11.47}$
Monocarboxylic acid metabolism	$10^{-8.99}$
Sulfur compound metabolism	$10^{-8.17}$
Organoic acid catabolism	$10^{-7.46}$
Nucleobase-containing small molecule metabolism	$10^{-5.92}$
Cholesterol homeostasis	$10^{-5.88}$
Peroxisome	$10^{-5.76}$
Valine, leucine and isoleucine degradation	$10^{-5.63}$
Bile acid & bile salt metabolism	$10^{-5.54}$
Lipid transport	$10^{-5.50}$
**Metascape pathway analysis: genes nearby CLP-specific HNF4α binding sites with increased expression 24h after CLP**	
**Description**	**P-value**
Response to growth factor	$10^{-6.60}$
Transcription by RNA polymerase II	$10^{-5.21}$
Transcription regulation from RNA polymerase II promoter in response to oxidative stress	$10^{-4.92}$
Asymmetric cell division	$10^{-3.77}$
Adipogenesis	$10^{-3.76}$
Negative regulation of protein processing	$10^{-3.14}$
Response to peptide	$10^{-3.14}$
Leukocyte homeostasis	$10^{-2.99}$
Canonical NFκB signal transduction	$10^{-2.96}$
Regulation of blood circulation	$10^{-2.91}$

Table S2 displays Metascape pathway analysis of downregulated (Padj < 0.05 and LFC < 0) or upregulated (Padj < 0.05 and LFC > 0) genes 24h after CLP associated with sham-specific (Padj < 0.05 and LFC < 0) or CLP-specific (Padj < 0.05 and LFC > 0) HNF4α binding sites, respectively, in liver 8h after sham or CLP. P-values derived from Fisher's exact test (Hypergeometric test). For differential analysis, P-values were calculated from DESeq2 (Wald test).

Related to figure 2.

Appendix Table S3. Pathway analysis ATAC-Seq 8h after CLP.

**Metascape pathway analysis: genes within sham-specific open chromatin sites and decreased expression 8h after CLP**	
Description	P-value
Regulation of GTPase activity	$10^{-9.07}$
Rho GTPase cycle	$10^{-8.30}$
Lipid metabolism	$10^{-7.16}$
Monocarboxylic acid metabolism	$10^{-6.81}$
Tube morphogenesis	$10^{-6.74}$
Nuclear receptors in lipid metabolism and toxicity	$10^{-5.58}$
Regulation of small GTPase mediated signal transduction	$10^{-5.57}$
Positive regulation of kinase activity	$10^{-5.32}$
Liver development	$10^{-5.28}$
Hippo signaling pathway	$10^{-5.20}$
**Metascape pathway analysis: genes within CLP-specific open chromatin sites and increased expression 8h after CLP**	
Description	P-value
Regulation of epithelial cell migration	$10^{-11.24}$
Cytokine signalling in immune system	$10^{-10.81}$
Egfr1 signaling	$10^{-10.52}$
Prositive regulation cytokine production	$10^{-10.30}$
Mapk signaling	$10^{-9.84}$
Signaling by receptor tyrosine kinases	$10^{-9.57}$
Actin filament organization	$10^{-7.80}$
Positive regulation of programmed cell death	$10^{-7.57}$
Positive regulation of response to external stimulus	$10^{-7.26}$
Regulationg of MAPK cascade	$10^{-7.24}$

Table S3 displays Metascape pathway analysis of downregulated (Padj < 0.05 and LFC < 0) or upregulated (Padj < 0.05 and LFC > 0) genes 8h after CLP associated with sham-specific (Padj < 0.05 and LFC < 0) or CLP-specific (Padj < 0.05 and LFC > 0) open chromatin sites, respectively, in liver 8h after sham or CLP. P-values derived from Fisher's exact test (Hypergeometric test). For differential analysis, P-values were calculated from DESeq2 (Wald test).
Related to figure 3.

Appendix Table S4. Pathway analysis ATAC-Seq 24h after CLP.

**Metascape pathway analysis: genes nearby sham-specific open chromatin sites with decreased expression 24h after CLP**	
Description	P-value
Monocarboxylic acid metabolism	$10^{-38.39}$
Lipid metabolism	$10^{-25.47}$
Steroid metabolism	$10^{-18.41}$
Amino acid metabolism	$10^{-15.91}$
Sulfur compound metabolism	$10^{-15.70}$
Biosynthesis of cofactors	$10^{-15.68}$
Biological oxidations	$10^{-15.42}$
Nucleobase-containing small molecule metabolism	$10^{-13.84}$
Bile secretion	$10^{-12.92}$
Lipid localization	$10^{-12.39}$
**Metascape pathway analysis: genes nearby CLP-specific open chromatin sites with increased expression 24h after CLP**	
Description	P-value
Positive regulation of locomotion	$10^{-11.07}$
Egfr1 signaling	$10^{-9.90}$
Signaling by Rho GTPases, Miro GTPases and RHOBTB3	$10^{-9.53}$
Signaling by receptor tyrosine kinases	$10^{-9.06}$
Regulation of plasma membrane bounded cell projection organization	$10^{-8.51}$
Actin filament-based process	$10^{-8.41}$
Regulation of cell secretion	$10^{-8.39}$
Negative regulation of intracellular signal transduction	$10^{-8.16}$
Mapk signaling	$10^{-8.07}$
Regulation of Mapk cascade	$10^{-7.63}$

Table S4 displays Metascape pathway analysis of downregulated (Padj < 0.05 and LFC < 0) or upregulated (Padj < 0.05 and LFC > 0) genes 24h after CLP associated with sham-specific (Padj<0.05 and LFC < 0) or CLP-specific (Padj < 0.05 and LFC > 0) open chromatin sites, respectively, in liver 8h after sham or CLP. P-values derived from Fisher's exact test (Hypergeometric test). For differential analysis, P-values were calculated from DESeq2 (Wald test).
Related to figure 3.

Appendix Table S5. Pathway analysis IL6 Hnf4a$^{Liver\text{-}i\text{-}KO}$ RNA-Seq.

**Top 10 pathways showing reduced IL6 induction in Hnf4a$^{Liver\text{-}i\text{-}KO}$: IPA**	
Description	P-value
Acute phase response	$10^{-15.136}$
Role of macrophages, fibroblasts & endothelial cells in RA	$10^{-7.741}$
Death receptor signaling	$10^{-7.131}$
LXR/RXR activation	$10^{-7.095}$
IL-10 signaling	$10^{-7.022}$
IL-6 signaling	$10^{-6.882}$
Hepatic cholestasis	$10^{-6.868}$
Hepatic fibrosis signaling	$10^{-6.174}$
GR signaling	$10^{-5.943}$
TLR signaling	$10^{-5.763}$
**Top 10 pathways showing increased IL6 induction in Hnf4a$^{Liver\text{-}i\text{-}KO}$: IPA**	
Description	P-value
Spliceosomal cycle	$10^{-6.396}$
tRNA charging	$10^{-5.241}$
Protein ubiquitination pathway	$10^{-3.816}$
miRNA biogenesis	$10^{-3.777}$
EIF2 signaling	$10^{-2.97}$
Cell cycle: G1/S checkpoint regulation	$10^{-2.836}$
Colanic acid building blocks biosynthesis	$10^{-2.731}$
Cell cycle: G2/M DNA damage checkpoint regulation	$10^{-2.682}$
ID1 signaling	$10^{-2.417}$
Cyclins & cell cycle regulation	$10^{-2.346}$

Table S5 displays IPA pathway analysis of IL6 target genes (defined by Padj < 0.05 in Hnf4a^{flfl} IL6 relative to PBS) that are less induced (LFC < 0) or more induced (LFC > 0) by IL6 in Hnf4a$^{Liver\text{-}i\text{-}KO}$ relative to Hnf4a^{flfl}, and downregulated (LFC < 0) or upregulated (LFC > 0), respectively, in Hnf4a$^{Liver\text{-}i\text{-}KO}$ PBS relative to Hnf4a^{flfl} PBS. P-values derived from Fisher's exact test (Hypergeometric test). For differential analysis, P-values were calculated from DESeq2 (Wald test).
Related to figure 6.

Appendix Table S6. List of primers used for qPCR analysis

Gene	Forward primer (5'-3')	Reverse primer (5'-3')
A2m	AGATGGTGAGATTTCGTGTTGGTC	ACGGTCCTGCCTGATTCTGTA
Acox1	TAACTTCCTCACTCGAAGCCA	AGTTCCATGACCCATCTCTGTC
Apcs	AGACAGACCTCAAGAGGAAAGT	AGGTTCGGAAACACAGTGTAAAA
Gapdh	TGAAGCAGGCATCTGAGGG	CGAAGGTGGAAGAGTGGGAG
Hmgcs2	GAAGAGAGCGATGCAGGAAAC	GTCCACATATTGGGCTGGAAA
Hprt	AGTGTTGGATACAGGCCAGAC	CGTGATTCAAATCCCTGAAGT
Hpx	AGCAGTGGCGCTAAATATCCT	CCATTTTCAACTTCGGCAACTC
Ppara	AGAGCCCCATCTGTCCTCTC	ACTGGTAGTCTGCAAAACCAAA
Rpl	CCTGCTGCTCTCAAGGTT	TGGTTGTCACTGCCTCGTACTT
Saa1	GGAGTCTGGGCTGCTGAGAAAA	TGTCTGTTGGCTTCCTGGTCAG
Saa2	TGGCTGGAAAGATGGAGACAA	AAAGCTCTCTCTTGCATCACTG
Slc25a20	GACGAGCCGAAACCCATCAG	AGTCGGACCTTGACCGTGT
hGapdh	TCAAGATCATCAGCAATGCC	TGTGGTCATGAGTCCTTCCA
hPpib	AAGTCACCGTCAAGGTGTATTTT	TGCTGTTTTTGTAGCCAAATCCT
hAcsl3	ATGGAAAACCAACCTCATAGCAA	GCCATCCCAGTTATACCAGCAA
hCldn1	TCTGGCTATTTTAGTTGCCACAG	AGAGAGCCTGACCAAATTCGT
hCyp7a1	GAGAAGGCAAACGGGTGAAC	GGATTGGCACCAAATTGCAGA
hCyp8b1	ATTTGGATACCGTTCAGTGCAA	CAGAAGCGAAAGAGGCTGTC
hLdlr	TCTGCAACATGGCTAGAGACT	TCCAAGCATTCGTTGGTCCC
hNr1h3	CCTTCAGAACCCACAGAGATCC	ACGCTGCATAGCTCGTTCC
hNr1h4	AACCATACTCGCAATACAGCAA	ACAGCTCATCCCCTTTGATCC
hNr1i3	GATGCTGGCATGAGGAAAGAC	TTGCTCCTTACTCAGTTGCAC
hPpara	CGGTGACTTATCCTGTGGTCC	CCGCAGATTCTACATTCGATGTT
hSlc27a2	TTTCCGCCATCTACACAGTCC	CGTAGGTGAGAGTCTCGTCG
hThrb	GGCGCAGCACGTTGAAAAAT	CACATCATCATGGTCCAGATGG
hAcsl1	CTTATGGGCTTCGGAGCTTTT	CAAGTAGTGCGGATCTTCGTG
hCpt1a	ATCAATCGGACTCTGGAAACGG	TCAGGGAGTAGCGCATGGT
hCpt2	CATACAAGCTACATTTCGGGACC	AGCCCGGAGTGTCTTCAGAA
hEhhadh	AAACTCAGACCCGGTTGAAGA	TTGCAGAGTCTACGGGATTCT
hHadha	ATATGCCGCAATTTTACAGGGT	ACCTGCAATAAAGCAGCCTGG
hHmgcs2	GACTCCAGTGAAGCGCATTCT	CTGGGAAGTAGACCTCCAGG
hSlc25a20	GACCAGCCAAAACCCATCAG	AGAGGGTGACCGACGAACA

Appendix Table S7. List of primers used for ChIP-qPCR analysis

Gene	Forward primer (5'-3')	Reverse primer (5'-3')
Ppara	GCCCCTCTCATTCCGAACC	GGGTGCTTGGTTGTAGGTCC
Apoa2	CACACTTCCACCCCCGTATC	GAGGTACATTCGCTAGGCCC
Hes6	CTCGAGAATAGCGCTCCAGT	TCGTACTTTCCCCGGACTTTG
Ugt2b1	TGACTAGATGAGTCTGATGGTGT	ACAAACTTCGTGAACCTTGAGC
