# Supplementary figures and images for: A critical role for HNF4α in polymicrobial sepsis-associated metabolic reprogramming and death
Source: EMBO Mol Med. 2024 Sep 11;16(10):13. doi: 10.1038/s44321-024-00130-1 (PMC11473810; doi:10.1038/s44321-024-00130-1)

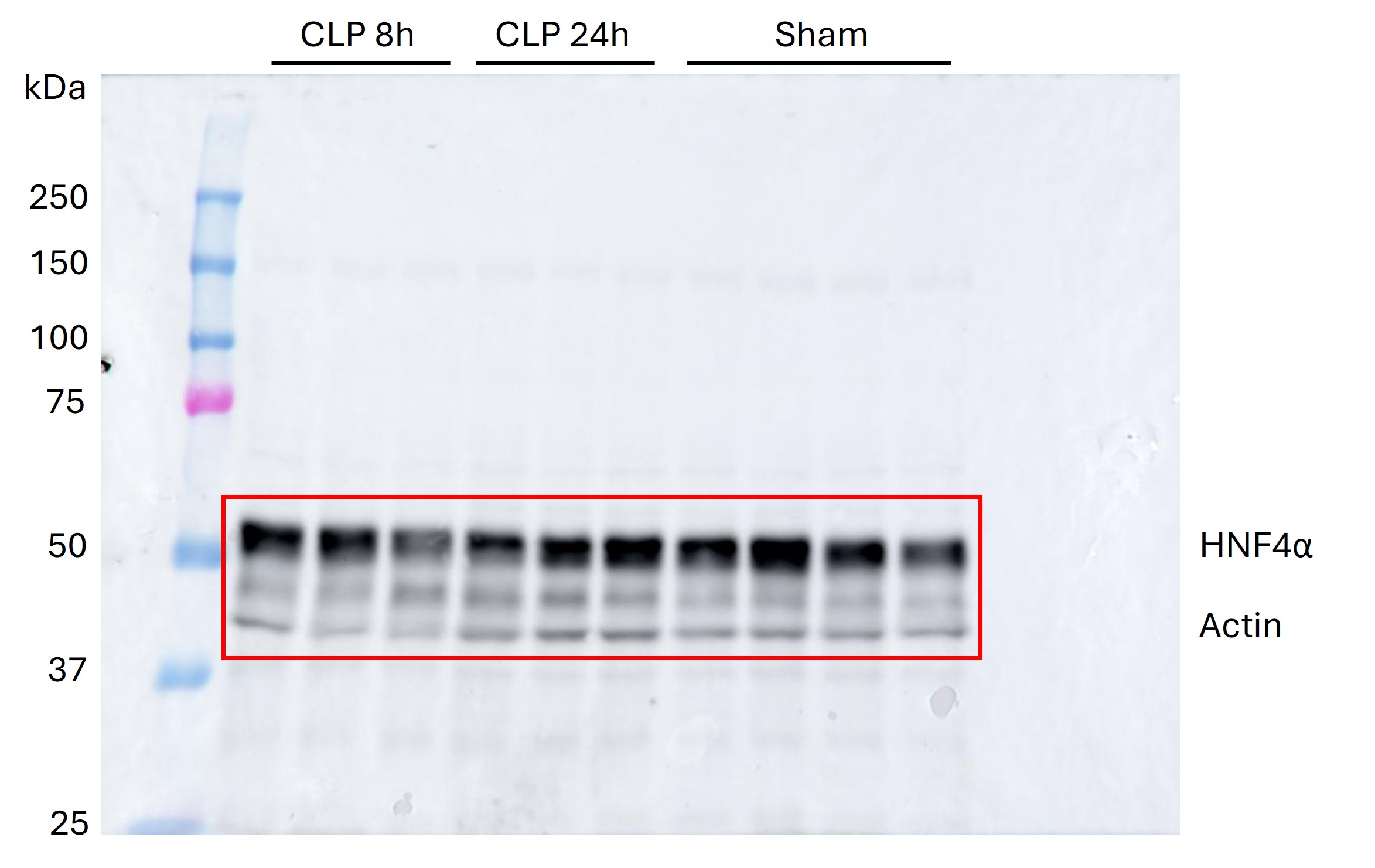

Supplement: Supplementary file 4 — Source data Fig. 2 [file 44321_2024_130_MOESM4_ESM.zip › Figure 2/2B/Western blot HNF4a+actin.jpg]

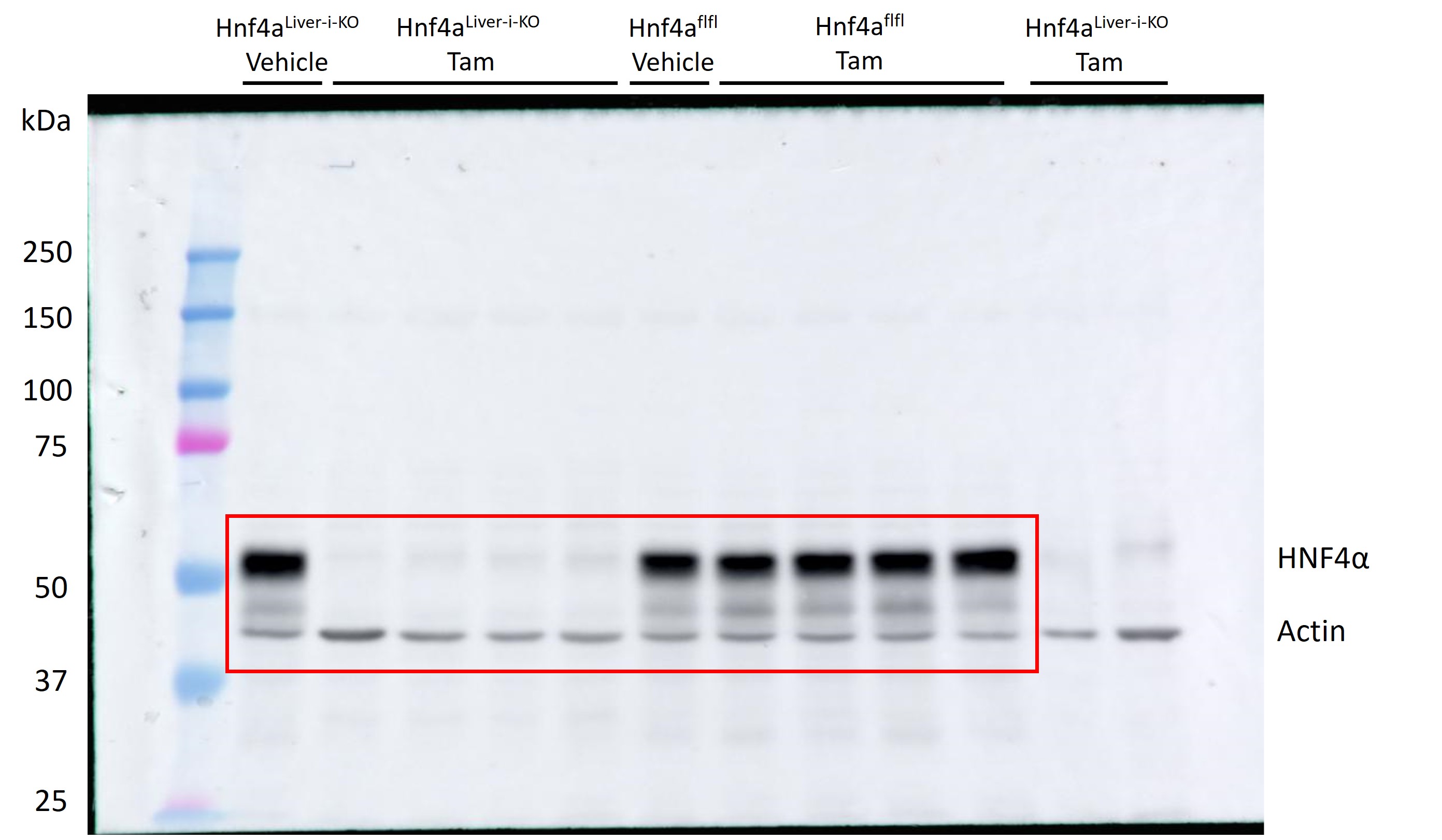

Supplement: Supplementary file 5 — Source data Fig. 4 [file 44321_2024_130_MOESM5_ESM.zip › Figure 4/4B/Western blot HNF4a+actin.jpg]
