# Supplementary material for: A critical role for HNF4α in polymicrobial sepsis-associated metabolic reprogramming and death
Source: EMBO Mol Med. 2024 Sep 11;16(10):13. doi: 10.1038/s44321-024-00130-1 (PMC11473810; doi:10.1038/s44321-024-00130-1)
Supplement: Supplementary file 9 — Expanded View Figures [file 44321_2024_130_MOESM9_ESM.pdf]

Expanded View Figures

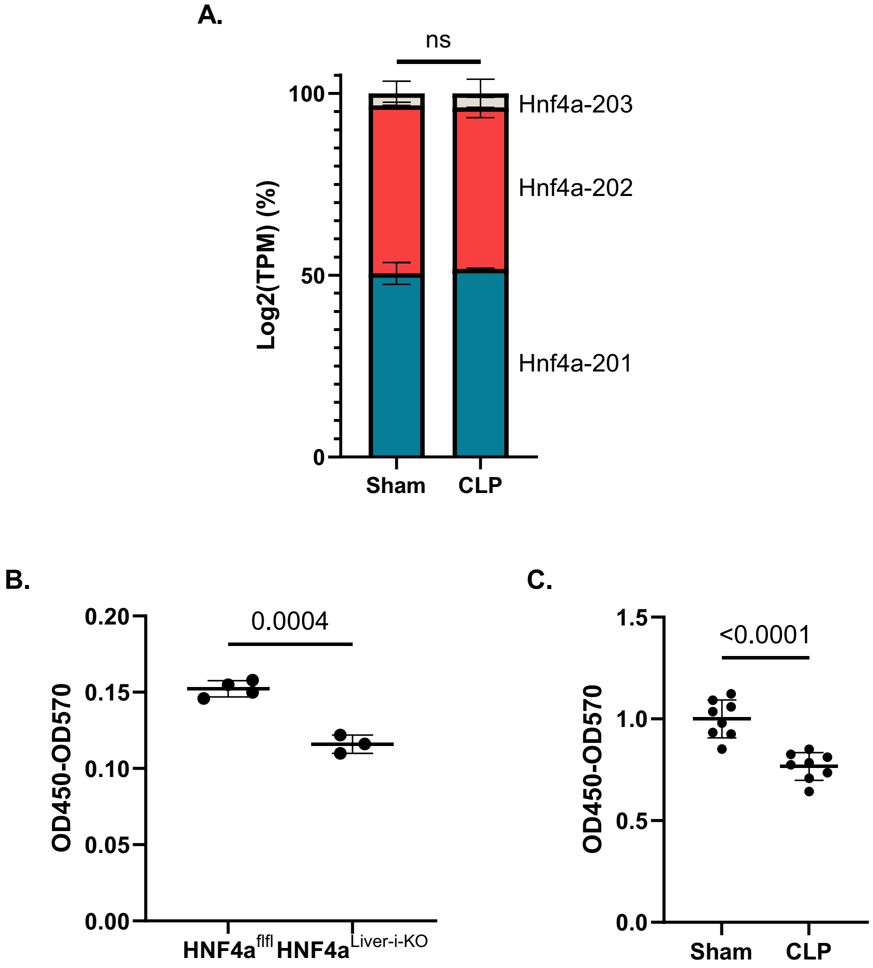

Figure EV1. Absence of alternative splicing with reduced HNF4α DNA binding in septic liver.

(**A**) Relative abundance of *Hnf4a* transcripts (Hnf4a-201, Hnf4a-202, and Hnf4a-203) in CLP relative to sham, expressed as log2(TPM) percentages, derived from in-house paired-end RNA-Seq data 8 h post-CLP. $n = 3$/group (biological replicates). (**B**, **C**) OD450-OD570 represents the strength of HNF4α binding to dsDNA oligos immobilized on a plate using nuclear lysates from tamoxifen-injected Hnf4a^{flfl} ($n = 4$) and Hnf4a$^{Liver\text{-}i\text{-}KO}$ ($n = 3$) mice (**B**) or sham ($n = 8$) and CLP ($n = 8$) mice 8 h post-CLP (**C**). Bars: mean ± SEM (**A**), central lines: mean ± SD (**B**, **C**). Each dot represents a single biological replicate. *P*-values were analyzed with two-way ANOVA (**A**) or unpaired t-test (**B**, **C**). ns: nonsignificant.

© The Author(s)

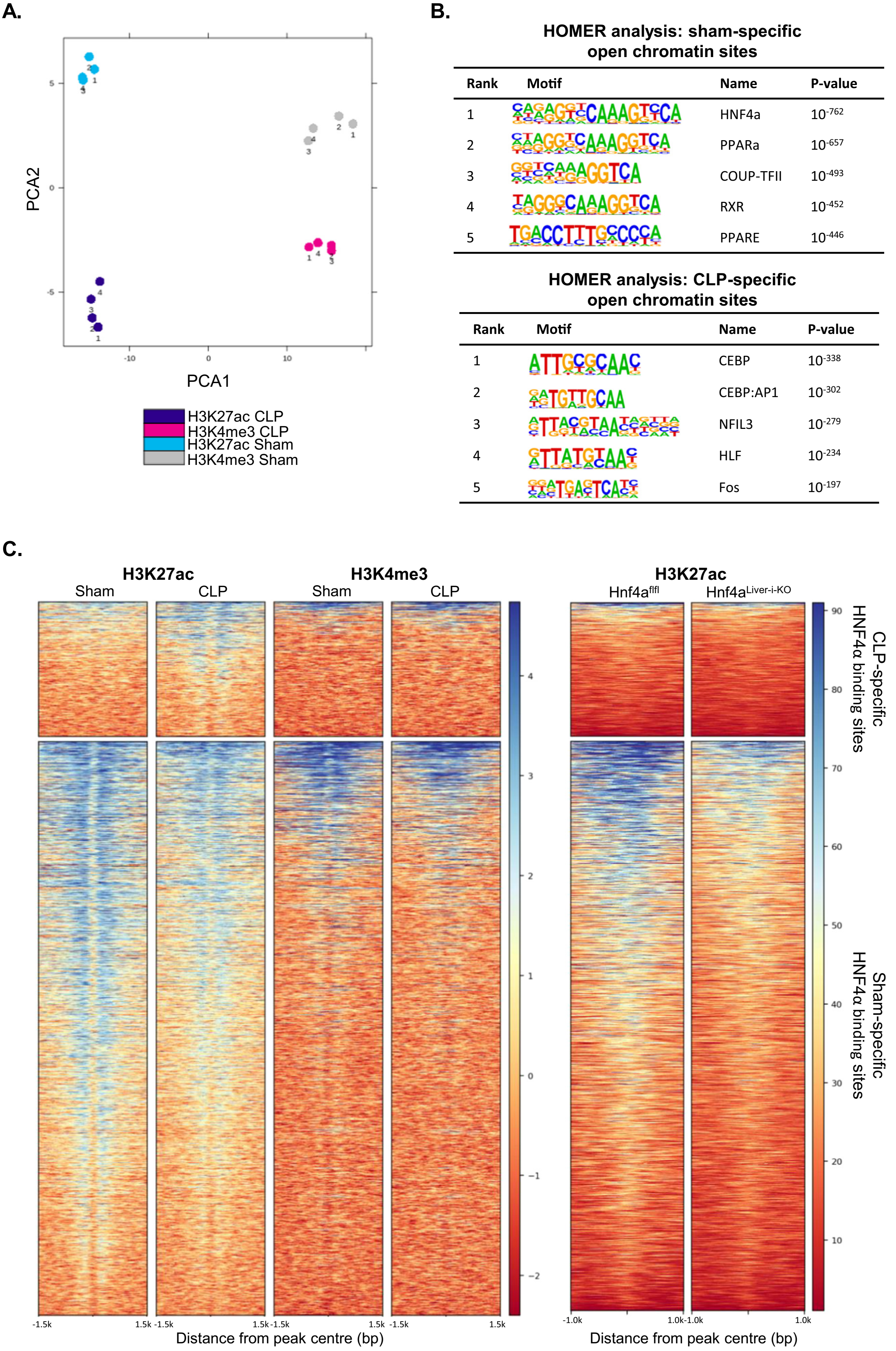
A.
PCA2
PCA1
5
0
-5
-10
0
10
H3K27ac CLP
H3K4me3 CLP
H3K27ac Sham
H3K4me3 Sham
B.
HOMER analysis: sham-specific open chromatin sites
Rank Motif Name P-value
1 HNF4a 10-762
2 PPARa 10-657
3 COUP-TFII 10-493
4 RXR 10-452
5 PPARE 10-446
HOMER analysis: CLP-specific open chromatin sites
Rank Motif Name P-value
1 CEBP 10-338
2 CEBP:AP1 10-302
3 NFIL3 10-279
4 HLF 10-234
5 Fos 10-197
C.
H3K27ac
Sham
CLP
H3K4me3
Sham
CLP
H3K27ac
Hnf4afl/fl
Hnf4aLiver-i-KO
CLP-specific HNF4α binding sites
Sham-specific HNF4α binding sites
-1.5k
1.5k
-1.0k
1.0k
Distance from peak centre (bp)

 © The Author(s)

Figure EV2. Perturbations in the chromatin binding dynamics of HNF4α during sepsis induce changes in the epigenetic landscape in the liver.

(**A–C**) Liver was isolated 8 h after sham or CLP for ATAC-Seq, and H3K4me3 and H3K27ac ChIP-Seq analyses. $n = 4$/group (biological replicates). (**A**) H3K4me3 and H3K27ac ChIP-Seq PCA plot. (**B**) HOMER transcription factor motif enrichment 200 bp centered on the center of all differential peaks. *P*-values derived from Fisher's exact test (Hypergeometric test). Differential peaks were identified by direct region overlap and DESeq2 (using Wald test), with the FDR set at 5%. (**C**) Heatmaps representing H3K27ac and H3K4me3 signals from sham and CLP or $Hnf4a^{fl/fl}$ and $Hnf4a^{Liver\text{-}i\text{-}KO}$ 1.5 kbp or 1.0 kbp, respectively, centered on the center of differential HNF4α ChIP-Seq peaks with decreased intensity ('sham-specific') and increased intensity ('CLP-specific') in CLP. Differential peaks were identified by DESeq2 (using Wald test), with the FDR set at 5%.

HNF4α-dependent pathways affected upon CLP

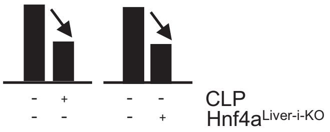

Pathways inhibited in Hnf4a^{flfl} CLP & Hnf4a$^{Liver\text{-}i\text{-}KO}$ sham: Enrichr	
**Description**	**P-value**
Estrogen response early	$1.077e^{-5}$
**Bile acid metabolism**	$4.695e^{-5}$
UV response Dn	$5.669e^{-3}$
**Cholesterol homeostasis**	$1.657e^{-2}$

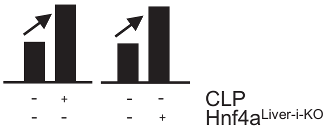

Pathways activated in Hnf4a^{flfl} CLP & Hnf4a$^{Liver\text{-}i\text{-}KO}$ sham: Enrichr	
**Description**	**P-value**
**Hypoxia**	$6.995e^{-6}$
Estrogen response early	$6.995e^{-6}$
**TNFα signaling via NFκB**	$3.784e^{-5}$
IL-2/STAT5 signaling	$1.791e^{-4}$
P53 pathway	$8.319e^{-4}$
Estrogen response late	$3.329e^{-3}$
E2F targets	$1.184e^{-2}$
Myc targets V1	$1.184e^{-2}$
KRAS signaling Up	$1.184e^{-2}$
Unfolded protein response	$1.275e^{-2}$
**Apoptosis**	$1.453e^{-2}$
Myc targets V2	$3.421e^{-2}$
G2-M checkpoint	$3.697e^{-2}$
Adipogenesis	$3.697e^{-2}$
**mTORC1 signaling**	$3.697e^{-2}$
**PI3K/AKT/mTOR signaling**	$4.026e^{-2}$

Figure EV3. HNF4α-dependent genes affected upon CLP function in distinct pathways.

Enrichr pathway analysis (MSIGDB Hallmark 2020) of genes significantly downregulated or upregulated (Padj < 0.05) in Hnf4a$^{Liver\text{-}i\text{-}KO}$ sham relative to Hnf4a^{flfl} (= HNF4α-dependent genes) and downregulated (Padj < 0.05, LFC < −0.8) or upregulated (Padj < 0.05, LFC > 0.8), respectively, in CLP relative to sham. *P*-values derived from Fisher's exact test (Hypergeometric test). Differential genes were identified by DESeq2 (using Wald test). The pathways we are mainly interested in, as evidenced by literature, are highlighted in bold.

 © The Author(s)

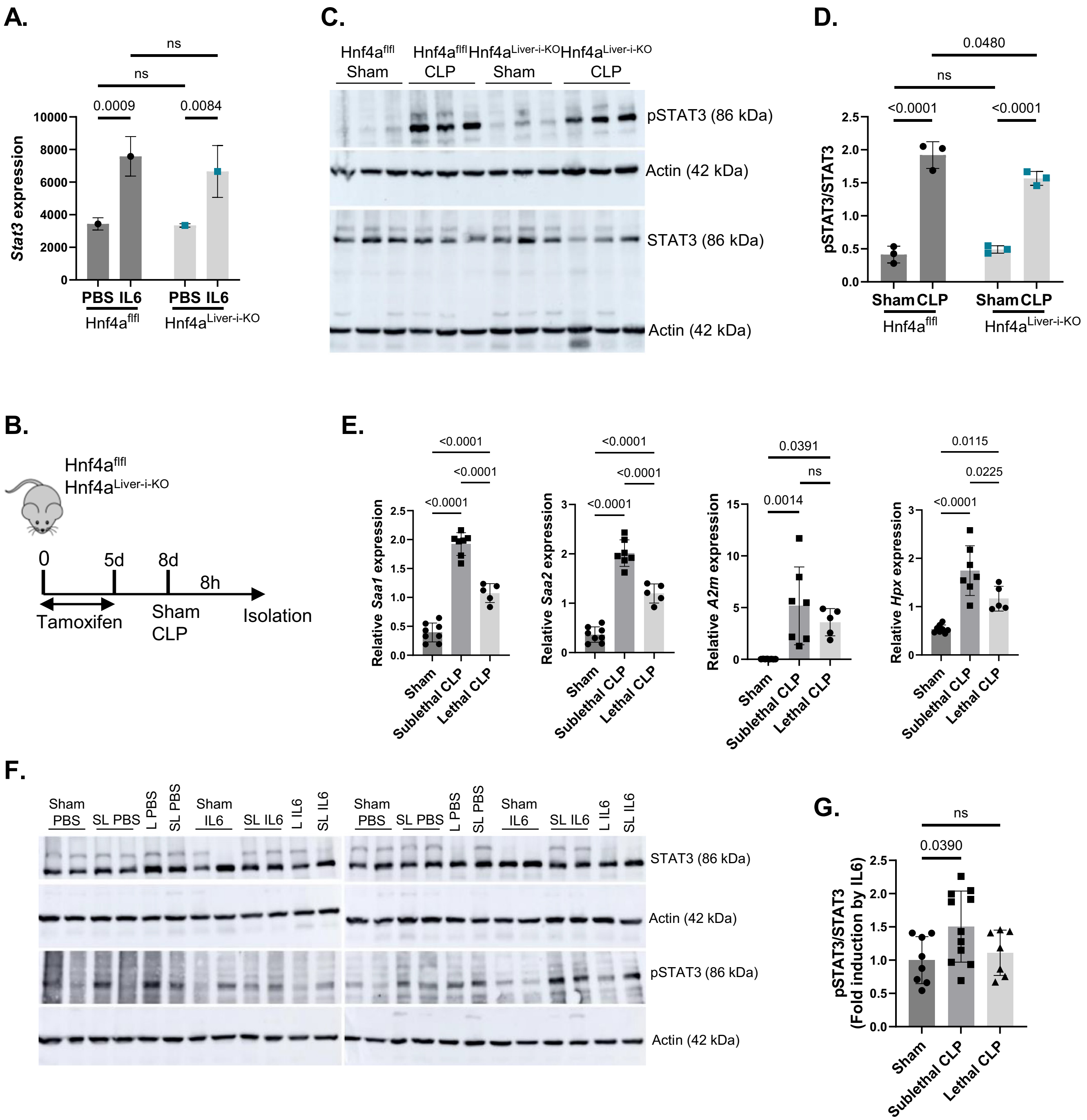

Figure EV4. Reduced STAT3 activity and hepatic acute phase response in Hnf4a^Liver-i-KO mice and in lethal sepsis.

(**A**) Hnf4a^Liver-i-KO and Hnf4a^flfl mice were treated with tamoxifen for 5 consecutive days, *i.p.* injected with hIL6 (100 µg/20 g) or PBS 3 days later, and liver was isolated 3 h later for RNA-Seq analysis. $n = 3$–4/group (biological replicates). Average normalized counts of *Stat3*. (**B–D**) Hnf4a^Liver-i-KO and Hnf4a^flfl mice were *i.p.* injected with tamoxifen on 5 consecutive days. Three days later, sham or CLP was performed, and livers were isolated 8 h later. (**B**) Experimental setup. (**C**, **D**) Western analysis of STAT3 (86 kDa) and phospho-STAT3 (Tyr705) (86 kDa) protein levels relative to actin (42 kDa). The ratio pSTAT3/STAT3 was determined as a measure for STAT3 activation. $n = 3$/group (6 female, 6 male). (**E**) RT-qPCR mRNA expression of *Saa1*, *Saa2*, *A2m*, and *Hpx* relative to *Hprt* and *Rpl* in livers from PBS-injected mice. $n = 5$–8/group. (**F**, **G**) Mice were *i.p.* injected with hIL6 (100 µg/20 g) or PBS 24 h after sham or CLP, and livers were isolated 3 h later. Sublethal (=SL) vs lethal (=L) CLP were distinguished by body temperature. Western analysis of STAT3 (86 kDa) and phospho-STAT3 (Tyr705) (86 kDa) protein levels relative to actin (42 kDa). The ratio pSTAT3/STAT3 was determined as a measure for STAT3 activation (fold induction by IL6). $n = 7$–11/group. Bars: mean ± SD. Each dot represents a single biological replicate. *P*-values were analyzed with two-way ANOVA (**A**), (**D**) or one-way ANOVA (**E**), (**G**). ns: non-significant.

A.

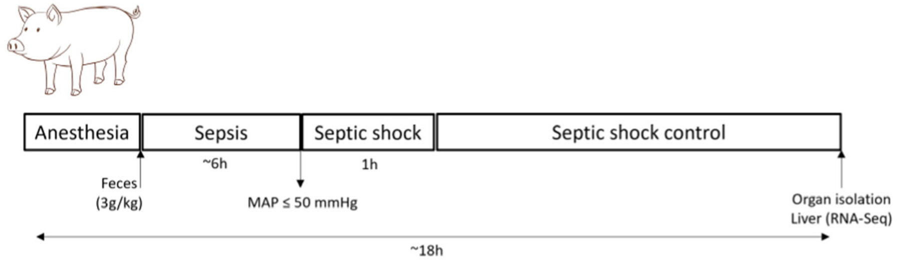

B.

Enrichr: downregulated genes in pig sepsis

	Name	P-value
**1**	**Hnf4a**	**3.083e-62**
**2**	Glis2	8.088e-11
**3**	Plagl2	9.755e-8
**4**	Creb1	4.782e-7
**5**	Esrra	1.992-6
**6**	Prdm1	9.110e-6
**7**	Tcof1	9.723e-6
**8**	Yy1	9.567e-5
**9**	Mist1	9.736e-5
**10**	Cdx2	1.304e-4

C.

HOMER motif analysis: downregulated genes in pig sepsis

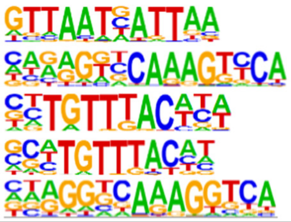

Rank	Motif	Name	P-value	# Target sequences with motif	% Target sequences with motif
1		HNF1b	1e-8	55	5,24%
2		**HNF4a**	**1e-8**	**147**	**14%**
3		FOXA2	1e-7	168	16%
4		FOXK2	1e-6	148	14,1%
5		PPARa	1e-6	293	27,9%

D.

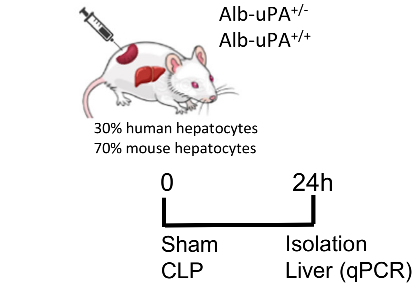

E.

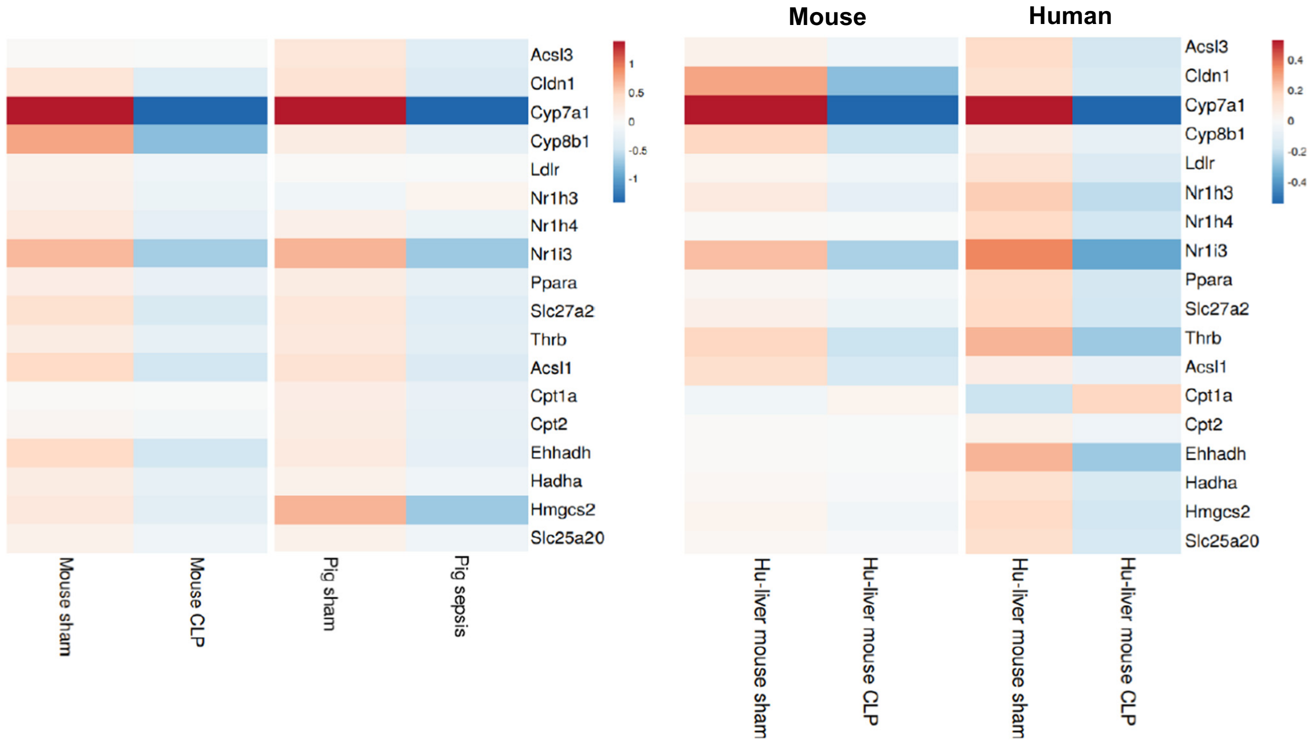

 © The Author(s)

Figure EV5. The relevance of HNF4α loss-of-function in porcine sepsis and in septic mice with humanized liver.

(**A**) Experimental setup porcine sepsis model. By intraperitoneal installation of 3 g/kg of autologous feces, the animals were permitted to develop sepsis until reaching a state of severe hypotension characterized by a mean arterial pressure (MAP) of ≤50 mmHg. Once severe hypotension, defined as a MAP ranging between 45 and 50 mmHg, was achieved, it was maintained for a duration of one hour. Around 18 h after sepsis initiation, liver samples were collected for RNA-Seq analysis ($n = 3$ sham, $n = 9$ sepsis; biological replicates). (**B**) Enrichr analysis of the genes downregulated (Padj < 0.05, LFC < 0) in pig sepsis. Differential genes were identified by DESeq2 (using Wald test). The upstream regulators are ordered by their enrichment *P*-value (derived from Fisher's exact test (Hypergeometric test)). (**C**) HOMER transcription factor motif enrichment 1000 bp upstream of TSS from the genes downregulated in pig sepsis. Differential genes were identified by DESeq2 (using Wald test). *P*-values were derived from Fisher's exact test (Hypergeometric test)). (**D**) At 2 weeks, Alb-uPA$^{+/+}$-SCID mice were injected with human hepatocytes into their spleens, enabling migration to the liver and repopulation of empty niches. After several weeks, mice underwent sham or CLP surgery, and livers were isolated 24 h later. (**E**) Heatmaps displaying the expression levels of HNF4α-dependent genes (genes differentially expressed in Hnf4a$^{Liver\text{-}i\text{-}KO}$ sham relative to Hnf4a$^{fl/fl}$ sham, with Padj < 0.05) in mouse liver 24 h post-CLP, pig sepsis liver and humanized mice liver 24 h post-CLP. We selected HNF4α target genes known to be downregulated (Padj < 0.05) in mouse liver 24 h post-CLP, focussing on the ones mentioned in the paper. Differential genes were identified by DESeq2 (using Wald test). Expression levels were quantified by RT-qPCR relative to *Hprt* and *Rpl*, and log transformed. For the humanized mice, human-specific and mouse-specific primers were used to investigate the response in human and mouse hepatocytes, respectively.

© The Author(s)
